# Ant genera identification using an ensemble of convolutional neural networks

**DOI:** 10.1371/journal.pone.0192011

**Published:** 2018-01-31

**Authors:** Alan Caio R. Marques, Marcos M. Raimundo, Ellen Marianne B. Cavalheiro, Luis F. P. Salles, Christiano Lyra, Fernando J. Von Zuben

**Affiliations:** 1 School of Electrical and Computer Engineering, University of Campinas (UNICAMP), Av. Albert Einstein 400, 13083-852 Campinas, São Paulo, Brazil; 2 Graduate Program in Ecology, Institute of Biology, University of Campinas (UNICAMP), R. Monteiro Lobato 255, 13083-862 Campinas, São Paulo, Brazil; University of South Carolina, UNITED STATES

## Abstract

Works requiring taxonomic knowledge face several challenges, such as arduous identification of many taxa and an insufficient number of taxonomists to identify a great deal of collected organisms. Machine learning tools, particularly convolutional neural networks (CNNs), are then welcome to automatically generate high-performance classifiers from available data. Supported by the image datasets available at the largest online database on ant biology, the AntWeb (www.antweb.org), we propose here an ensemble of CNNs to identify ant genera directly from the head, profile and dorsal perspectives of ant images. Transfer learning is also considered to improve the individual performance of the CNN classifiers. The performance achieved by the classifiers is diverse enough to promote a reduction in the overall classification error when they are combined in an ensemble, achieving an accuracy rate of over 80% on top-1 classification and an accuracy of over 90% on top-3 classification.

## Introduction

Taxonomy is a cornerstone for biodiversity management, since information on species names and distributions is essential for scientific studies and environmental monitoring programs [1]. However, biodiversity research currently faces a series of obstacles which hinder the development of works founded on taxonomic knowledge [2, 3]. Among these obstacles are the great number of specimens requiring identification and the concomitant low number of taxonomists available to perform this task [2, 4].

A suggested approach to deal with these issues is the use of artificial intelligence techniques [5, 6]. Although identification by experts should be the preferred way to identify specimens, computational intelligence systems may provide reliable alternative tools for taxonomic identification [2, 5, 7] while reducing the number of routine identifications performed by taxonomists [6]. A promising approach for this task is Deep Learning, particularly Convolutional Neural Networks (CNNs), a machine learning technique widely applied to image recognition [8]. Indeed, the use of CNNs for species identification from images has already been shown to be reliable for plants [9], aquatic macroinvertebrates [10], mammals [11], and insects [12–14].

Aiming at developing a reliable machine learning algorithm for quickly identifying a large amount of specimens, we developed a CNN automated ant genera identification process that receive as input the head, profile and dorsum perspectives of ant images. This dataset is a medium-size dataset; although CNNs is capable of automatically detecting discriminant attributes—which is essential here for a high-performance ant genera identification—it needs a large size dataset, and over-fitting can be an issue. In order to address the characteristics of this dataset, we proposed ensembles to aggregate the three image perspectives (head, dorsum and profile), and explored different types of training (general, specific and transfer learning). Such approach allows to deal with the possible issues arising from dataset sample size.

## Methods

### Dataset characterization

**Fig 1:**
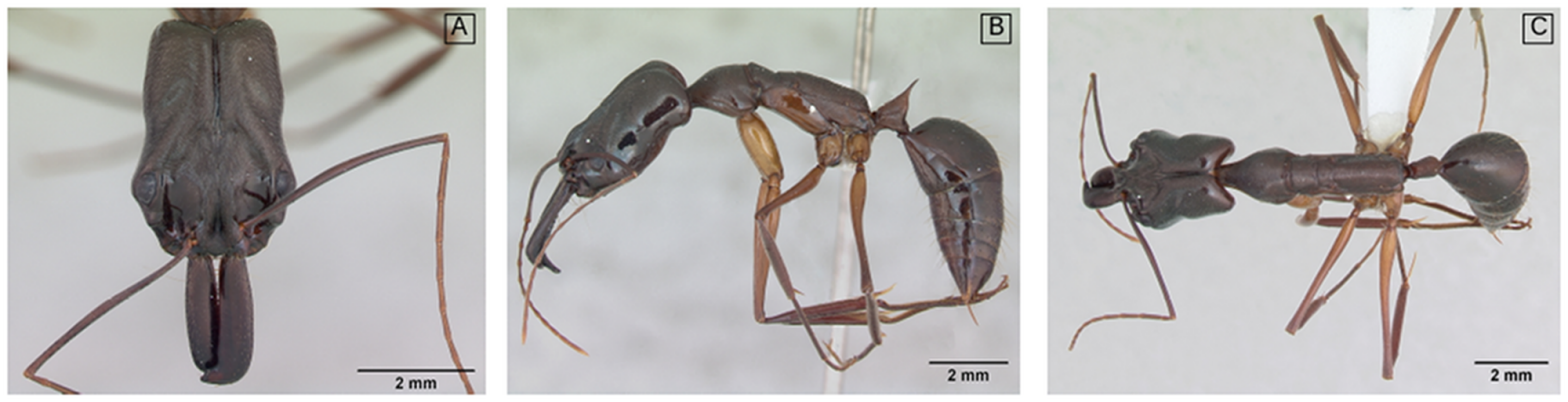
Example of head A, profile B, and dorsal C image perspectives of an *Odontomachus chelifer* specimen. Ant specimen photographs by April Nobile. Taken from www.antweb.org.

Ant identification requires mounting specimens on a piece of paper glued on a pin (Fig 1), so as to allow the observation of several morphological structures on the specimen’s head, dorsum and profile. Ant images deposited on databases therefore commonly consist of three views per specimen: a frontal, a lateral and a dorsal one (Fig 1).

We gathered ant images from AntWeb (www.antweb.org), the largest online database on ant biology, containing 44,806 specimens with at least 1 picture and an average number of 3.35 pictures per specimen, totalizing 150,088 pictures. Other relevant dataset characteristics are provided in Table 1.

**Table 1:** Summary of dataset characteristics. The columns show, respectively: the cardinality constraint; the number of specimens satisfying the constraint; and the rate of the data satisfying the constraint (first row determines the whole set). *N*_*h*_, *N*_*d*_ and *N*_*p*_ are, respectively, the cardinality of pictures from head, dorsum and profile perspective for each specimen.

Characteristic	# of specimens	Rate
*N*_*h*_ + *N*_*p*_ + *N*_*d*_ ≥ 1	44806	−
*N*_*h*_ = *N*_*p*_ = *N*_*d*_ = 1	35027	78%
*N*_*h*_ ≥ 1 and *N*_*p*_ ≥ 1 and *N*_*d*_ ≥ 1	42944	96%
*N*_*h*_ ≥ 1	43970	98%
*N*_*p*_ ≥ 1	44372	99%
*N*_*d*_ ≥ 1	43648	97%

We classified this dataset as being multi-view, because, when each specimen is considered as a sample, there are three views for each sample. Also, we classified this dataset as a medium size dataset because the major CNN reference dataset, the image-net.org, has above 1,000,000 images.

**Fig 2:**
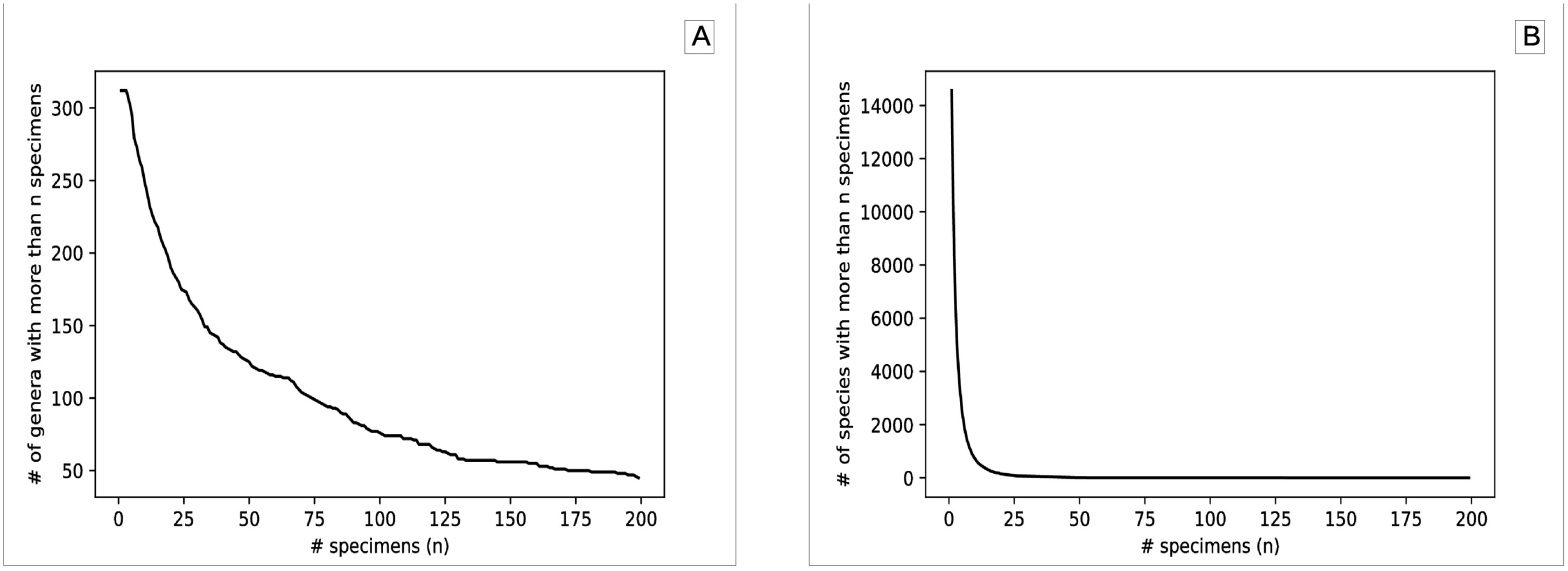
Number of ant genera A and species B with at least *n* specimens.

Multi-view methods that uses one picture per view may have difficulties with this dataset, because keeping specimens with only one picture per view (discarding the extra pictures) causes a reduction of 14% in the number pictures (resulting in 42,944 specimens and 128,832 pictures). Considering 100 as the reasonable minimum number of specimens for training CNN from scratch, we decided to identify ant genera, instead of ant species, because there are only two species achieving this criteria (Fig 2A), against *L* = 57 ant genera (Fig 2B).

The dataset was divided into three sets: 70% of the base was allocated for training the network; 20% of the dataset for validation; and 10% of the dataset for test. The validation and test datasets were composed only of specimens with exactly one image per view, which performed 78% of the total number of specimens cataloged. The training set is used to adjust the weights of the neural network. The validation set is used to evaluate the quality of specific purpose classifiers and the diversity of the models. After the training procedure, the best model in validation is the one selected for classification. The test set is then used to evaluate the quality of the proposed classifier.

The nature of the dataset allows one to conclude that each image sample *p* of a specimen *s* from a perspective *v* ∈ {*h*, *d*, *p*} has a labeled genus *g* ∈ {1, …, *L*}. Two kinds of learning procedures are investigated: 1) given a picture sample *p* of any specimen or perspective, classify the label *l*; 2) given a specimen *s* with one picture of each perspective *v* ∈ {*h*, *d*, *p*}, classify the label *l*. On evaluation, both methods deliver a list with the *t* most likely classes for each sample, called top-*t*. Therefore, the prediction is considered correct if the correct class is within the obtained list.

Given the structure of our data, the following hypotheses were investigated:

1. We expect that the dataset is large enough to train a CNN for image prediction (Type 1), ensuring a high-performance general-purpose classifier (capable of classifying an image from any perspective).
2. We expect that a specific-purpose classifier (capable of classifying images from a specific perspective) has a fair performance compared to the general-purpose classifier, but a specific-purpose classifier built using transfer learning knowledge from the model trained for general-purpose is capable of outperforming both.
3. Since the images are taken from different perspectives, we expect that, the classification pattern of each perspective of a specimen will lead to accurate models, with reasonable consensus on most of the data, yet preserving diversity and thus helping to predict more difficult samples.
4. If premise 3 is true, an ensemble aggregating the outputs of each classifier (from different perspectives and types of training) should improve the classification performance.

The Type 2 classifier applied to scenarios with multiple data perspectives is commonly treated in the machine learning literature as a multi-view learning problem. The main challenge of this field is to explore the multitude of data without overfitting, which could happen when the method concatenates the data features. This behavior can be avoided by constructing learners that integrate features without directly sharing them. Although the main methods to deal with this problem are co-training, multiple kernel learning and subspace learning, there are some similarities with ensemble learning [15]. Still, ensemble is applied to multi-view data by aggregating the votes of random forests trained to each view [16]. Multi-view CNN approaches involve appending the multiple views as channels [17], using a fine-tune ImageNet-trained CNN for each view, for concatenating the penultimate layer output for each view, training a classifier on top of it [18] and simultaneously training a CNN for which the initial layers are view-specific convolutional layers [19].

Many multi-view insights are contemplated by our proposal, despite the fact that this multi-view approach is mainly based on a simple ensemble aggregation. The general purpose classifier tries to explore the common features through all views; the specific purpose classifier creates individual classifiers per view; and the transfer learning classifier tries to find a specific view regularized by the common features through all views. These approaches can explore many characteristics of the data, and the final classifier produced by the aggregation of all views benefits from this diversity.

In the **Convolutional Neural Network Architecture** section we describe the Type 1 (single-view) CNN classifier and the architecture employed in this work, and in section **CNN Training procedure** we describe the train procedure applied to training the general and specific purpose CNNs. Also, the **CNN Training procedure** section, describes experiments devoted to validating hypotheses 1 and 2. The **Ensemble** section describes some ensemble properties and metrics and evaluate the ensemble behavior to access the correctness of hypothesis 3. In the **Ensemble** section we also use the general and specific purpose classifiers to construct a Type 2 classifier using an ensemble aggregation. To check hypothesis 4, we evaluate the performance of single classifiers and compare them with the ensembles.

The experimental setup do gather the dataset, train the different CNNs, and build the ensemble is available at dx.doi.org/10.17504/protocols.io.kzpcx5n.

## Convolutional neural network architecture

Convolutional neural networks (CNNs) are characterized by a neural network with many information processing steps, also known as layers. In a CNN, the *i*th layer transforms the previous feature vector, consisted of *c*_*i* − 1_ channels with *n*_*i* − 1_ × *n*_*i* − 1_ cells, in another feature vector consisted of *c*_*i*_ channels with *n*_*i*_ × *n*_*i*_ cells.

**Fig 3:**
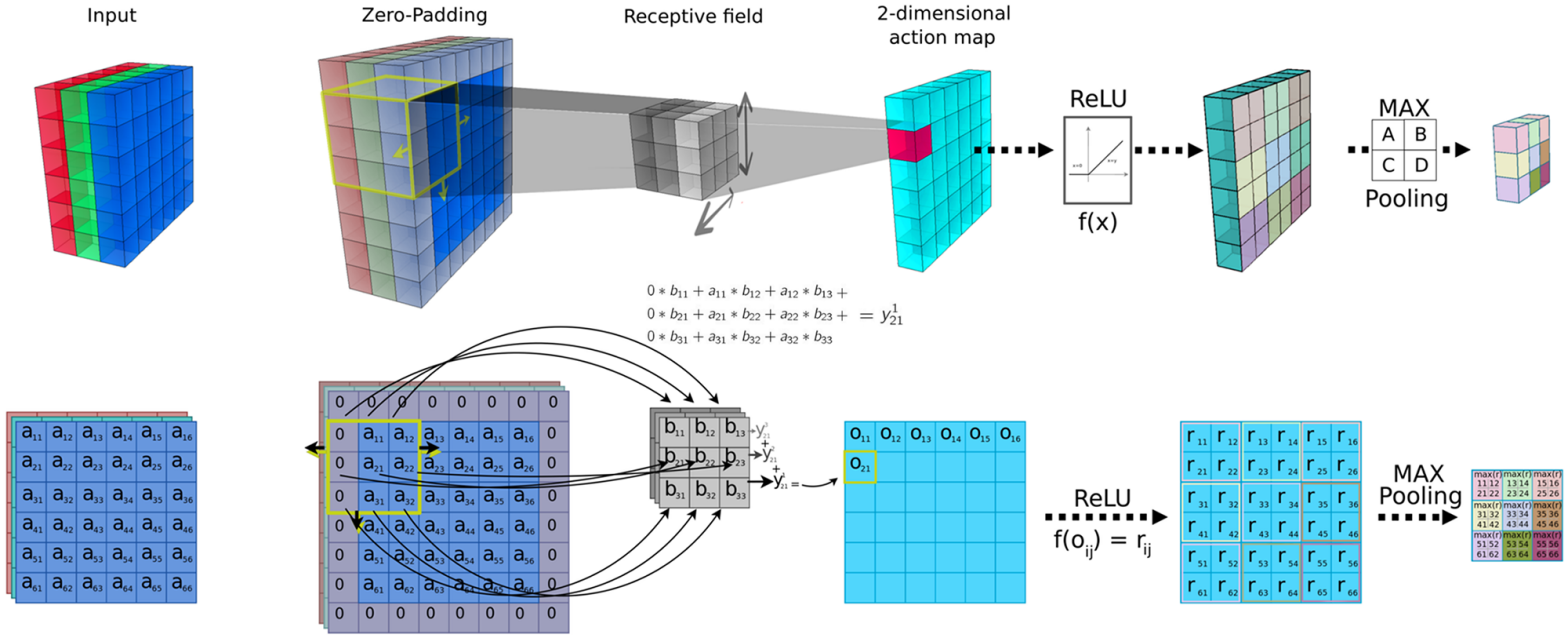
Operation in a convolutional layer with ReLU and Pooling. The first column shows a (6 × 6) input example with 3 channels. The columns from second to forth show the interaction of this input with a receptive field (3 × 3, represented in the third column) generating a (6 × 6) output with 1 channel in the forth column. The second column shows a padding with value 1, and the applied stride is also 1. The fifth column represents the output of a ReLU operation. The last column presents the 3 × 3 output for a 2 × 2 window Max Pooling operation (represented in the sixth column).

The description of each type of layer is given as follows [20], and the linkage between consecutive layers can be seen in Fig 3:

**Convolution** - Considering a previous layer with *c*_*i* − 1_ channels with *H*_*in*_ × *W*_*i*_ cells, a convolution layer is formed by the interaction of this layer with a number of receptive fields (also called filters). The quantity of filters, *C*_*out*_, are windows with *c*_*i* − 1_ channels and *k* × *k* weights, and each window will scroll the whole bidimensional output of the previous layer with the same set of filters. This scroll can be performed in different ways, controlled by two variables, padding and stride.

Padding or Zero-padding is the insertion of extra values around the bidimensional input. The extra values are generally set to zero, so as to not change the value of the input. This information can help the receptive field to work better at the borders of the matrix or to control the dimension of the output.

Stride is the shift step of the receptive fields throughout the matrix of pixels. With stride equal to 1, the shift is of one pixel.

The operation of the receptive field over the output of the previous layer results in *C*_*out*_ bidimensional action maps, each one with dimension *H*_*out*_ × *W*_*out*_, which can be calculated by:

$$\begin{array}{c} H_{out}=\frac{H_{in}-k+2P}{S}+1 \end{array}$$ (1)

*W*_*out*_ is generally taken to be equal to *H*_*out*_; *C*_*out*_ is the number of receptive fields with dimension *k* × *k*, *P* is the padding and *S* is the stride.

**Pooling** - A pooling operation calculates a specific norm of the activation values produced by a small region of each channel. The goal of this calculation is to decrease the computational costs by reducing the size of each channel of the previous layer.

**Normalization** - Used to accelerate the convergence process and reduce the odds of being stuck in a local minimum. This procedure normalizes the output layer signals using linear and non-linear operations.

**Fully connected** - Each cell of the output in this layer is calculated by making a weighted sum of all cells from all channels of the previous layer.

**Dropout** - Apply a transformation which is similar to a fixed mask that makes a cell as zero (saving the cell for future use) for half of the cells (this rate can be changed). Thus, the calculation of the gradient is ignored by these cells during some epochs. After this period, the saved values of the reset cells are recovered and another random mask is applied, reseting half of the cells at random. The objective here is to avoid overfitting by reducing co-adaptation.

**ReLu** - The activation function is defined by *f*(*x*) = *max*(0, *x*). This is a linear by parts function that accelerates weight adjustment, when compared to traditional sigmoidal functions.

**Softmax** - It is the final operator at the output of the neural network. The output for each label is exponentiated and then divided by the sum of the output of all the labels in order to make this output vary between zero and one, expressing the degree of membership of the analyzed sample to each class.

**Loss** - It is the performance index that calculates the classification error rate.

The relationship between the calculated outputs and the expected results are used to generate a cost function that is to be implements the iterative. The optimization technique used is the stochastic gradient descending, which adjustment of the weights.

The **network architecture** employed in this work was based on the AlexNet [21], but using the Caffe framework [22]. The input layer receives an image with dimension 256x256 in gray-scale.

**Fig 4:**
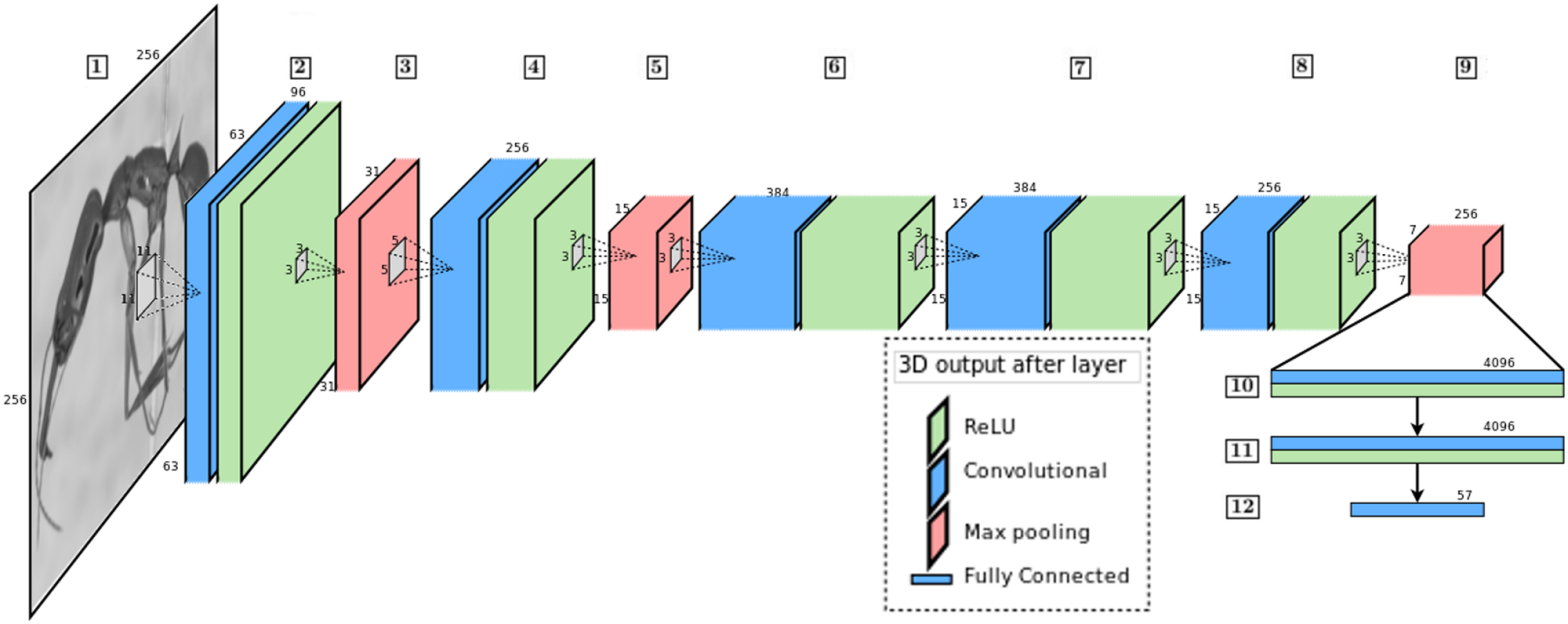
CNN architecture used to perform image classification. Ant specimen photograph by April Nobile, taken from www.antweb.org.

The sequence of layers, presented in Fig 4, is enumerated in what follows:

1 Input in grayscale—256 × 256.
2 Convolution with 11 × 11 receptive field, *stride* = 4: Output 63 × 63 × 96 with ReLU in sequence.
3 Pooling with 3 × 3 receptive field, *stride* = 2: Output 31 × 31 × 96 with Normalization Layer in sequence.
4 Convolution with values to keep dimensions: Output 31 × 31 × 256 with ReLU in sequence.
5 Pooling with 3 × 3 receptive field, *stride* = 2: Output 15 × 15 × 256 with Normalization Layer in sequence.
6 Convolution with values to keep dimensions: Output 15 × 15 × 384 with ReLU in sequence.
7 Convolution with values to keep dimensions: Output 15 × 15 × 384 with ReLU in sequence.
8 Convolution with values to keep dimensions: Output 15 × 15 × 256 with ReLU in sequence.
9 Pooling with 3 × 3 receptive field, *stride* = 2: Output 7 × 7 × 256.
10 Fully connected and dropout—output: 4096 channels.
11 Fully connected and dropout—output: 4096 channels.
12 Fully connected softmax and loss—output: 57 channels.

## Constructing an ensemble of CNN for multi-view ant genera identification

### CNN training procedure

First of all, we created a **general purpose classifier**. The training procedure (for 50000 epochs) for this learning machine uses the training set to calculate the classification loss. Then, back-propagation is applied to obtain the gradient vector that will guide the weight adjustment of the neural network. The validation error is monitored throughout the training epochs to keep the most accurate model. As described in the **Convolutional Neural Network Architecture** section, given that the samples of this training came from any view, this machine was trained to be capable of classifying the genus of an ant using any image perspective.

Since distinct features can be observed in each image view, we decided to make specific learning machines for each of them, and we called these machines **specific purpose classifiers**. In order to do this, we split the dataset by image view and performed the same training procedure (for 50000 epochs) of the general one, but creating one machine for each view. Since we have one third of the base for each view and this shortage could cause overfitting we propose a kind of **transfer learning** strategy to deal with this. This strategy consists in creating one learning machine for each view by loading the already trained **general purpose classifier** and continuing the training for 30000 epochs with images from one view only. This whole process generates three new classifiers, one for each view, in order to extract the specific features of each view.

In order to provide baseline performance values against our approach, we extracted the outputs from the penultimate layer of an AlexNet CNN trained with the image-net.org dataset and used such outputs as feature vectors for a SVM training [18]. The SVM training procedure used the SVC classifier (SVM object for classification, version 0.18.1) of scikit-learn.org, keeping all parameters in the default values, with the exception of the regularization strength—this parameter was tuned with the hyperopt library (available at github.com/hyperopt), which searches the regularization strength with best accuracy on the validation set of a SVC model trained in the training set. The performance values correspond to the validation set of the most accurate model.

Tables 2 and 3 give the accuracy, average precision for each label and the minimum label performance on precision per label for the top lists; Table 2 shows the baseline methods and Table 3 shows the proposed methods. We choose precision as the main metric because it is more frequently used by biologists than recall, and it is relevant to achieve a high precision for all genera. Accuracy has a widespread use in machine learning, and it also complements the precision metrics. Hypothesis 1 turned to be true because the general purpose methods were satisfactory, with at least 90% of accuracy and average precision on top-3 and top-5 lists, and at least 80% of minimum precision on top-3 and top-5 lists. We can also verify hypothesis 2 by observing a small performance drop of the specific purpose classifier, but this behavior is reverted in the specific purpose with transfer learning, even outperforming the general purpose one. Both hypothesis are strengthened when compared with the baseline performances; Hypothesis 1 is reinforced because, with more data (in the general case), the performance is consistently better than the baseline performance (except in some cases in the minimum precision metric); this is not the case when the data is scarce (in the specific case). Hypothesis 2 is also reinforced because the transfer learning has a performance that is similar to the obtained in the general case, also consistently better than the baseline performance.

**Table 2:** Classification performance of the SVM with linear and rbf kernel, when the features are extracted from the penultimate layer of an AlexNet CNN trained with an www.image-net.org dataset. Rows show the performance of each learning machine (SVM with linear kernel and SVM with rbf kernel) on each image view (head, dorsum and profile). Columns show accuracy, average precision and minimum precision performance for each label on top lists. H = head view; D = dorsal view; P = profile view; SVM-L = SVM with linear kernel; SVM-R = SVM with rbf kernel.

		Accuracy			Average precision			Minimum precision		
		Top 1	Top 3	Top 5	Top 1	Top 3	Top 5	Top 1	Top 3	Top 5
H	SVM-L	0.69	0.87	0.92	0.67	0.88	0.93	0.38	0.75	0.86
	SVM-R	0.71	0.87	0.92	0.68	0.88	0.92	0.39	0.77	0.87
D	SVM-L	0.55	0.76	0.84	0.52	0.77	0.84	0.00	0.64	0.77
	SVM-R	0.57	0.77	0.84	0.53	0.78	0.85	0.22	0.70	0.78
P	SVM-L	0.55	0.76	0.84	0.49	0.76	0.84	0.14	0.62	0.77
	SVM-R	0.58	0.77	0.85	0.51	0.77	0.85	0.13	0.67	0.77

**Table 3:** Classification performance of the CNN learning machines in the validation set. Rows show the performance of the learning machines (general, specific and transfer) on each image view (head, dorsum and profile). Columns show accuracy, average precision and minimum precision performance for each label on top lists. H = head view; D = dorsal view; P = profile view; G = general classifier; S = specific classifier; T = transfer classifier.

		Accuracy			Average precision			Minimum precision		
		Top 1	Top 3	Top 5	Top 1	Top 3	Top 5	Top 1	Top 3	Top 5
H	G	0.78	0.91	0.94	0.73	0.90	0.94	0.46	0.81	0.88
	S	0.78	0.90	0.94	0.72	0.90	0.94	0.33	0.83	0.88
	T	0.78	0.91	0.94	0.74	0.91	0.94	0.28	0.83	0.90
D	G	0.62	0.80	0.86	0.54	0.79	0.86	0.15	0.58	0.76
	S	0.56	0.75	0.82	0.50	0.74	0.82	0.14	0.57	0.69
	T	0.60	0.79	0.86	0.54	0.78	0.86	0.11	0.55	0.74
P	G	0.66	0.83	0.89	0.57	0.81	0.88	0.19	0.64	0.77
	S	0.61	0.79	0.85	0.50	0.77	0.84	0.12	0.57	0.72
	T	0.67	0.84	0.89	0.58	0.83	0.88	0.23	0.65	0.80

### Ensemble

Since we have three perspectives of images, we hoped to find a divergent but accurate prediction for different views of the same specimen, giving rise to one of the main properties required to construct an ensemble: diversity [23]. We also investigated two multi-view properties: consensus principle, which relates performance to the agreement of multiple views; and complementary principle, which states that each view can provide an information not contained in other views, and is related to diversity in the components of the ensemble [15]. Since the models were already generated, we implemented filtering selection and an aggregation procedure to create an ensemble.

#### Diversity

In order to verify the agreement and diversity (complementarity) present in the trained ensembles, we adopted pairwise metrics comparing two classifiers based on double-fault measures [24]: **both correct measure (bc)**, the rate of samples for which both classifiers are correct; **both fault measure (df)**, the rate of samples for which both classifiers are incorrect; **some correct measure (sc)**, the rate of samples for which at least one classifier is correct; and **only one correct measure (oc)**, the rate of samples for which only one classifier is correct. The **sc** indicates the potential for accurate performance, since it counts the proportion of at least one of the classifiers in the pairwise evaluation being correct. The **oc** measure can be interpreted as the useful diversity, which indicates the number of disagreements in the classifiers that can potentially increase the performance, or as an indicator of complementarity. From a multi-view perspective, **bc** can be seen as a consensus metric and **ec** as a complementary metric.

Given these pairwise measures, a global performance of an ensemble is given by the average of the computed diversity on all pairwise combinations of the ensemble components. Table 4 shows these diversities considering four types of ensembles: 1) aggregating components associated with each view in the general purpose classifier **(G)**; 2) aggregating components associated with each view in the specific purpose classifier **S**; 3) aggregating components associated with each view in the transfer learning **T**; 4) aggregating all views and all classification methods **All**. We can see that hypothesis 3 is true because all ensembles have a good diversity measure, achieving high values of **sc**. The ensemble *All* has the highest performance on *oc* and an excellent performance on **sc**, which show that this ensemble has diverse components, potentially achieving a good classifier performance. Under a multi-view perspective, the aggregation of general classifiers shows good consensus and the aggregation of specific classifiers guides to good complementary classifiers. This behavior is expected, but any extreme solution can be harmful. To solve this issue, a transfer learning approach is implemented, presenting a good trade-off between both principles, and the final aggregation of all approaches also shows a good performance in deference to the multi-view perspective.

**Table 4:** Diversity performance on sets of learning machines on validation set. G = general classifier; S = specific classifier; T = transfer classifier.

	Top 1			Top 3			Top 5		
	sc	bc	oc	sc	bc	oc	sc	bc	oc
G	0.84	0.72	0.12	0.94	0.88	0.06	0.96	0.92	0.04
S	0.68	0.51	0.17	0.84	0.71	0.13	0.90	0.80	0.10
T	0.72	0.57	0.15	0.87	0.77	0.10	0.91	0.84	0.08
All	0.81	0.54	0.27	0.92	0.74	0.18	0.96	0.82	0.13

#### Classifier

Using the models trained in **CNN Training procedure** section and the diversity analyses of the **Diversity** section, it is possible to construct an ensemble. Following the steps needed to build an ensemble [23], we constructed four types of ensembles.

**Fig 5:**
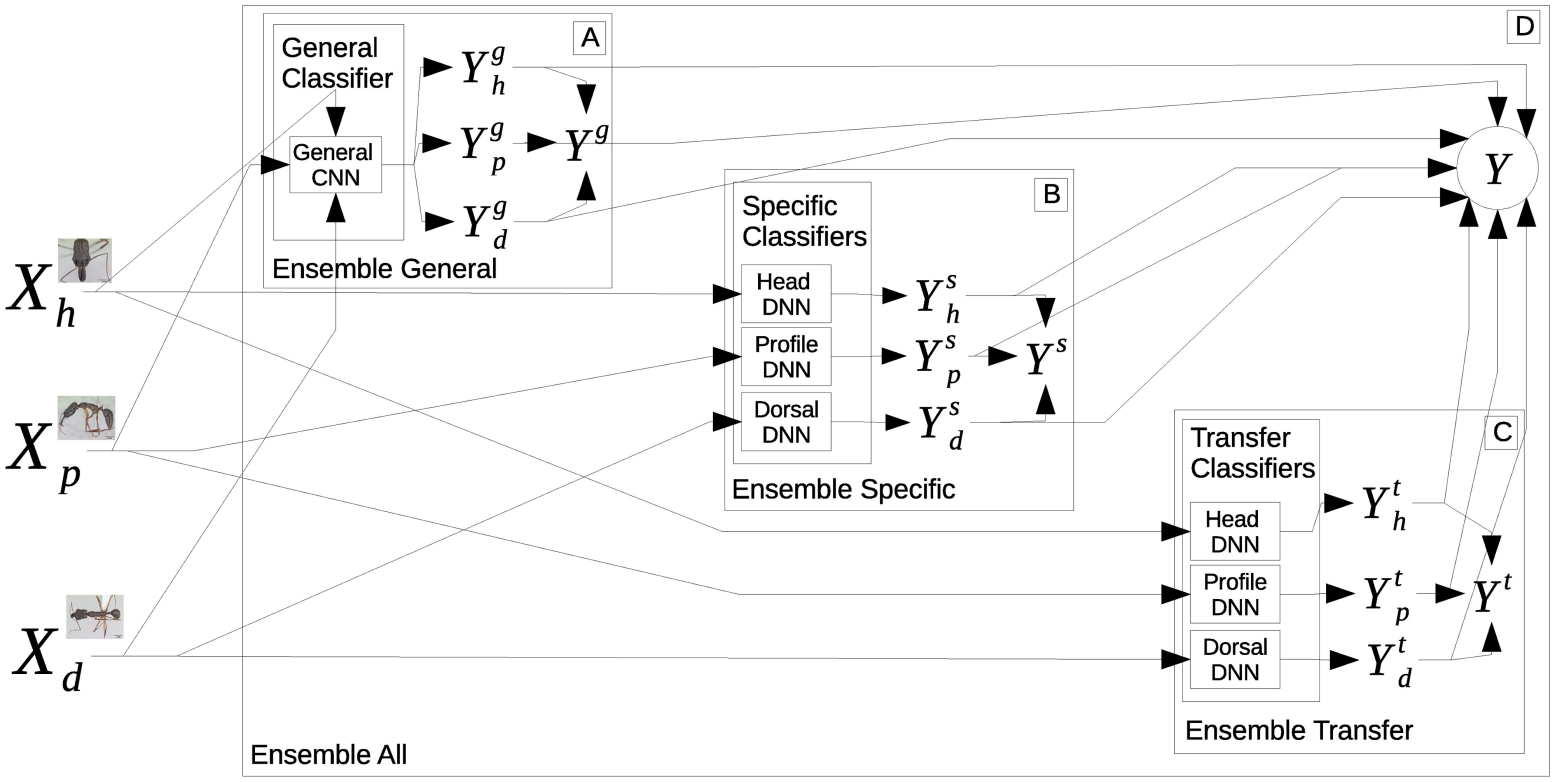
Representation of the ensemble exploring different types of image views. Ant specimen photographs by April Nobile, taken from www.antweb.org.

The first one is conceived by aggregating all general and specific purpose classifiers and summing up the output distributions [25]. This ensemble is represented in Fig 5D, where the final output is given by a summation of all outputs $Y=\sum_{m\in \{g,s,t\}}\sum_{v\in \{h,p,d\}}Y_{v}^{m}$.

The other three ensembles are made by summing up only the machines generated by the **general** purpose $Y^{g}=\sum_{v\in \{h,p,d\}}Y_{v}^{g}$ (represented in Fig 5A), by the simple **specific** purpose $Y^{s}=\sum_{v\in \{h,p,d\}}Y_{v}^{s}$ (represented in Fig 5B) or by the **transfer** specific purpose $Y^{t}=\sum_{v\in \{h,p,d\}}Y_{v}^{t}$ (represented in Fig 5C). Performances for these ensembles and single predictors (CNNs) are shown in Table 5.

**Table 5:** Classification performance of ensembles and CNN learning machines in the test set. The best ensemble and single view classifiers for each performance metric are highlighted in bold and blue, respectively. H = head view; D = dorsal view; P = profile view; G = general classifier; S = specific classifier; T = transfer classifier; E = ensemble classifier.

		G			S			T			E			
		H	D	P	H	D	P	H	D	P	G	S	T	All
Accuracy	Top 1	0.77	0.61	0.64	0.76	0.54	0.60	0.78	0.59	0.65	0.81	0.79	0.81	**0.83**
	Top 3	0.91	0.80	0.82	0.90	0.74	0.78	0.91	0.78	0.83	0.92	0.90	0.92	**0.93**
	Top 5	0.94	0.87	0.88	0.94	0.82	0.85	0.94	0.85	0.89	0.95	0.94	0.95	**0.96**
Average precision	Top 1	0.74	0.53	0.54	0.72	0.46	0.51	0.74	0.52	0.57	0.80	0.78	0.79	**0.83**
	Top 3	0.91	0.79	0.81	0.89	0.74	0.77	0.91	0.77	0.81	0.92	0.91	0.92	**0.94**
	Top 5	0.94	0.86	0.87	0.94	0.83	0.84	0.95	0.85	0.89	0.95	0.95	**0.96**	**0.96**
Minimum precision	Top 1	0.32	0.11	0.24	0.31	0.00	0.20	0.30	0.16	0.24	**0.45**	0.39	0.43	0.40
	Top 3	0.76	0.54	0.58	0.79	0.56	0.60	0.79	0.53	0.64	0.81	0.81	0.83	**0.84**
	Top 5	0.86	0.74	0.74	0.86	0.70	0.73	0.89	0.69	0.81	0.89	0.91	**0.92**	0.90

Table 5 indicates that hypothesis 4 is true, because almost all ensembles have better performance compared to the single machines, in all metrics for all types of lists (top-1, top-3 and top-5). The only exception is the ensemble of specific purpose, which is worse than a machine for the head perspective in general purpose, when top-3 accuracy is considered.

## Discussion

We developed an efficient learning system based on CNNs to identify ant genera from available datasets. The system explores the diversity of multiple perspectives (head, dorsum and profile) from the same sample (an ant specimen) to create an ensemble that aggregates the classification of CNNs for each perspective.

For almost all classifiers the best prediction rates were achieved using head views. We find two possible explanations for this. Classification from head views may be more accurate because the position of the specimen’s legs in profile and dorsal views might add more noise to the picture than the position of the antennae in the head view (see Fig 1). Another possible explanation is that ant genera may have more pronounced morphological differences in the head due to greater selective pressures for niche differentiation in this body part. For instance, eye size in ants may be related to several ecological pressures such as navigation and capture of prey; antennae size may influence the detection of chemosensory stimuli; and mandible shape may be determinant for prey capturing and handling [26]. Indeed, ant genera have already been shown to be morphologically differentiated mostly by body size (which our system does not take into account) and morphological characters in the head [27], [26].

The ensemble classifier we developed exhibited promising performance in predicting ant genera from images. Even though the classification of a single perspective showed a good performance mainly for the average precision and accuracy, classifiers of distinct perspectives have also shown diversity, thus motivating the usage of ensemble aggregation. Indeed, all metrics used in the top-1 classification were improved by the ensembles, as were the top-3 and top-5 minimum precision. This classifier achieved over 80% for both accuracy and average precision on top-1 and over 90% of accuracy and average precision on top-3 and top-5, showing a really good performance in the average use of the system. Even in the prediction of the most challenging genus, the ensemble guarantees a fair performance on top-1 (40%) and a great performance on top-3 (84%) and top-5 (90%) lists, which demonstrates high confidence and robustness in ant genera identification.

The performance achieved by the transfer learned single view classifiers, mainly for head and profile perspectives, brings insights both for transfer learning and multi-view applied to CNN classifiers: The transfer learning strategy recovered the lost performance when used to train the network from scratch, with better results for all metrics compared to the specific purpose classifiers. It also increased performance compared to the general purpose classifier for all metrics in head and dorsum perspective, achieving the best results for almost all cases when used on head perspective images. Given that, considering images with the same dimension, training a general purpose classifier (CNN capable of classifying images from any view), followed by a transfer learning strategy to specify the classification of each view, can be a simple and straightforward way to create a classifier which improves the generalization performance by looking for other view attributes.

In summary, the paper proposed an efficient learning system based on CNNs to identify ant genera. We believe our work contributes towards extracting taxonomic knowledge without human intervention. The work also contributes to the Machine Learning field, bringing new insights on the usage of ensembles for multi-view structured data, and on the transfer learning mechanisms to explore shared attributes on multi-view CNNs.
